# Annual Meeting of the Constituents

**Published:** 1918-12-28

**Authors:** 


### METROPOLITAN HOSPITAL SUNDAY FUND.

### Annual Meeting of the Constituents.

The Annual Meeting of the Constituents of the Metropolitan Hospital-Sunday Fund was held at the Mansion House, on Friday, December 20. The Right Honourable Sir Horace Brooks Marshall, Lord Mayor (President and Treasurer) occupied the chair, and among those present were Sir John Bell, Rev. J. G. Curry, Mr. R. McConnell, Mr. Edward Dent, Mr. Edward Eyre, Rev. A. A. Harland, Sir George Makins, G.C.M.G., C.B., F.R.C.S., Mr. J. H. Morgan, F.R.C.S., Mr. Edwin Pollock, Rev. Prebendary Reynolds, Lord Somerleyton, Sir Marcus Samuel, Sir Charles Wakefield, Rev. Mgr. Carton de Wiart.

**The Secretary** (Mr. Arnold James) read the notice convening the meeting, and announced that he had received letters regretting inability to be present from Sir E. Tritton, the Archdeacon of London, the Chief Rabbi, Sir Henry Burdett, K.C.B., Sir A. Pearce Gould, M.S., K.C.V.O., Sir Dyce Duckworth, Rev. J. A. Mayo, Mr. J. E. Moxey, and Mr. J. H. Buxton (Hon. Secretary).

On the motion of the President it was agreed that the annual report be taken as read.

**Sir Marcus Samuel,** in moving the adoption of the report, said he felt sure it was a matter of very deep regret that Sir Ernest Tritton was unable to be present owing to indisposition. It would be quite impossible to over-rate the splendid work he had done on behalf of the Fund. The total receipts for the year were £92,055, being £15,701 in excess of the previous year, and many thousands in excess of any figures previously reached. It was most gratifying to know that the good impulses of the charitable had not only not been arrested by the progress of the war, but appeared to have been stimulated by it. The church collections amounted to £43,077, being £5,739 more than last year. Donations were £13,257, an increase of £5,106, due to the handsome gift of £5,000 from the American Red Cross in recognition of the great work done by London hospitals for the wounded. Great thanks were due to the clergy and other ministers for the notable increase in the church collections, largely on account of the extension by them of the plan of carrying the call of the hospitals into the homes of the people. Reports had been received from clergy and ministers who had tried the plan, and it was agreed that good resulted from it, not only to the hospitals but to the churches. Moreover, the fund was a great help to the clergy in their parochial and social work. On the whole, the experiment had proved so great a success that they sincerely hoped its scope would be enlarged, and that every clergyman would do his utmost to carry out the scheme of home solicitation. The sum distributed among the hospitals, dispensaries, and nursing associations was £85,038, in addition to which grants had been made amounting to £2,973 in respect of 6,892 surgical appliances for the poor patients recommended by the clergy and ministers. Their thanks were due, and were most heartily given, to the Press for very helpful co-operation, and to Messrs. Harrods, Peter Robinson, and Selfridge for so generously placing part of their advertising space at the disposal of the Fund during the week preceding Hospital Sunday. A point for business men to note was that, notwithstanding the great increase in the cost of printing, stationery, postage, upon which the work of the Fund so largely depended, the percentage of the cost of collection and administration was lower than it had ever been. He could not sit down without saying how much they owed to Mr. James, their secretary, for the excellent results obtained in many ways.

**Rev. P. Oakley Hill** seconded the resolution, and expressed appreciation of the satisfactory increase in the Fund for the year. It had far exceeded the high-water mark of £80,181 for the year 1908. Some people imagined that during the progress of a war the fountains of generosity dried up, but the very opposite had been the case. He believed that the British public had learned that wonderful maxim of Our Lord enshrined in the Acts of the Apostles: "It is more blessed to give than to receive." Again, it was satisfactory to know that there had been an increase of £5,000 in the collections in various places of worship, a considerable portion of which sum was collected in poor parishes of London. For instance, in his own parish of Hoxton there was received this year more than eight times the amount collected there six years ago. That had been the experience of many of his brother clergy, and could probably be accounted for by the new method of collecting. Whereas some years ago they only collected from their congregations, they now went out into the parishes, and found how very ready the poor were to give of their pence, if only they had the opportunity, in order that they might support what was probably the most popular Fund in London. Therein lay encouragement to hope that the high-water mark reached during the current year would be exceeded next year, and that the collection would amount to £100,000. If they set that goal before them and did their best to reach it, he felt sure they would succeed. He also would like to bear testimony to the effective work done by Mr. James, who, as Secretary of the Fund, devoted all his energies and thoughts to its advancement.

The Report was unanimously adopted.

**Rev. O. R. Dawson** moved the re-election of the Council with the exception of the names of Rev. Canon Gamble, Rev. Canon Allen Bell, Rev. Prebendary Swayne, and with the addition of the names of Rev. E. Marling Roberts, Rev. W. G. Pennyman, Rev. Dr. Jowett and Sir Charles Hanson, Bt., to fill the vacancies. He felt certain that there would be no criticism of the motion, for the Council had served them well during the past year. He could remember when there had been very spirited debates during the progress of the annual meeting, but there could be no dissatisfaction in regard to the accounts for the past year, and they were therefore very grateful to the Council for the work they had done.

**The Rev. G. C. Wilton** seconded the resolution which was carried unanimously.

**Lord Somerleyton** moved that Hospital Sunday, 1919, be fixed for June 22. He thought next year would prove as great a record as the one just ended. A great many people would probably be ready to offer some sort of thanksgiving for the end of the terrible war. In any event, it was matter for the warmest congratulation to them all that the Fund had made such a magnificent record in the past year, when the King's Fund had also been enabled to distribute a larger amount than ever before, and he trusted the Saturday Fund had been in a like position.

**Sir William Church** seconded the motion, and said that the decision to fix a day for Hospital Sunday which did not vary, as in former years, with the Church's calendar had proved most satisfactory. He hoped that next year they would more nearly approach what had been the ambition of their Treasurer and many others—namely, the distribution of the sum of £100,000.

**The President,** in acknowledging a vote of thanks to himself for presiding, said it had been a very great pleasure and honour to preside over the meeting. It was true that the year had been a record one in many ways. We were on the fringe of what he hoped and believed would be a satisfactory peace, and he was sure that nothing could be more fitting than that in such a year—a year which could, at any rate, be described, if as nothing else, as a victory year—there should be record collections and subscriptions for the Hospital Sunday Fund.

